# Editorial: The 16th annual *Nucleic Acids Research* web server issue 2018

**DOI:** 10.1093/nar/gky518

**Published:** 2018-06-22

The 2018 web server issue of *Nucleic Acids Research* is the 16th in a series of annual issues dedicated to web-based software resources for analysis and visualization of molecular biology data. It is freely available online under *NAR*’s open access policy. This year, 273 proposals were submitted and 99, or 36%, were approved for manuscript submission. Of those approved, 85 or 86%, were ultimately accepted for publication. Table 1 lists the 2018 web servers, their URLs, and a brief description of each.

**Table 1.** Descriptions of web servers in the *NAR* 2018 web server issue

Web server name	URL	Brief description
**AAI-profiler**	http://ekhidna2.biocenter.helsinki.fi/AAI	proteome average amino acid identity comparison
**AlloFinder**	http://mdl.shsmu.edu.cn/ALF/	allosteric modulator identification
**ArDock**	http://ardock.ibcp.fr	protein–protein interaction region prediction
**BAGEL4**	http://bagel4.molgenrug.nl	secondary metabolite gene clusters (RIPPs, bacteriocins)
**BaMM**	https://bammmotif.mpibpc.mpg.de	nucleotide binding motifs
**BeStSel**	http://bestsel.elte.hu	circular dichroism spectroscopy based protein secondary structure analysis
**BRepertoire**	http://mabra.biomed.kcl.ac.uk/BRepertoire	antibody repertoire analysis
**BUSCA**	http://busca.biocomp.unibo.it	protein subcellular localization prediction
**CABS-flex 2.0**	http://biocomp.chem.uw.edu.pl/CABSflex2	simulation of protein structure flexibility
**CalFitter**	https://loschmidt.chemi.muni.cz/calfitter/	protein thermal denaturation analysis
**CASTp 3.0**	http://sts.bioe.uic.edu/castp/	topology of protein pockets, cavities and channels
**CavityPlus**	http://www.pkumdl.cn/cavityplus	protein binding site cavities
**CellAtlasSearch**	http://www.cellatlassearch.com	single cell gene expression data search
**cgDNAweb**	http://cgDNAweb.epfl.ch	double-stranded DNA coarse-grain models
**CircadiOmics**	http://circadiomics.ics.uci.edu	circadian rhythm dataset analysis and repository
**COACH-D**	http://yanglab.nankai.edu.cn/COACH-D/	protein–ligand binding site prediction
**Coloc-stats**	https://hyperbrowser.uio.no/coloc-stats/	genomic location enrichment analysis
**ComplexContact**	http://raptorx2.uchicago.edu/ComplexContact/	protein heterodimer complex residue–residue contact prediction
**CoNekT-Plants**	http://conekt.plant.tools	comparative analyses of plant gene co-expression
**CRISPOR**	http://crispor.org	guide sequences for CRISPR/Cas9 genome editing
**CRISPRCasFinder**	https://crisprcas.i2bc.paris-saclay.fr	CRISPR array and Cas gene detection
**CSAR-web**	http://genome.cs.nthu.edu.tw/CSAR-web	contig scaffolding
**dbCAN2**	http://cys.bios.niu.edu/dbCAN2	carbohydrate-active enzyme annotation
**DynaMut**	http://biosig.unimelb.edu.au/dynamut/	point mutation effects on protein stability and dynamics
**easyFRAP-web**	https://easyfrap.vmnet.upatras.gr/	protein mobility analysis with fluorescence recovery after photobleaching data
**EviNet**	https://www.evinet.org/	gene set network enrichment analysis
**ezTag**	http://eztag.bioqrator.org	biomedical concept annotation
**FragFit**	http://proteinformatics.de/FragFit	protein segment modeling of cryo-EM density maps
**Freiburg RNA tools**	http://rna.informatik.uni-freiburg.de	RNA analysis
**GADGET**	http://gadget.biosci.gatech.edu	population-based distributions of genetic variants
**Galaxy**	https://usegalaxy.org	biomedical data analysis workflows
**Galaxy HiCExplorer**	https://hicexplorer.usegalaxy.eu	chromatin 3D conformation analysis
**GDA**	http://gda.unimore.it/	integration of drug response, gene expression profiles and mutations for cancer
**GeneMANIA**	http://genemania.org	gene function prediction
**geno2pheno[ngs-freq]**	http://ngs.geno2pheno.org	viral drug resistance prediction
**GIANT 2.0**	http://giant-v2.princeton.edu	human tissue-specific gene functional relationships
**GPCRM**	http://gpcrm.biomodellab.eu/	G protein-coupled receptors structure modeling
**gRINN**	http://grinn.readthedocs.io	protein molecular dynamics residue interaction energies
**GWAS4D**	http://mulinlab.org/gwas4d	prioritization of regulatory variants from GWAS data
**HMMER**	http://www.ebi.ac.uk/Tools/hmmer	profile hidden Markov models homology search
**HotSpot Wizard 3.0**	http://loschmidt.chemi.muni.cz/hotspotwizard3	protein engineering directed mutation
**HPEPDOCK**	http://huanglab.phys.hust.edu.cn/hpepdock/	peptide–protein docking
**HSYMDOCK**	http://huanglab.phys.hust.edu.cn/hsymdock/	symmetric protein complex docking
**InterEvDock2**	http://bioserv.rpbs.univ-paris-diderot.fr/services/InterEvDock2/	protein–protein docking
**INTERSPIA**	http://bioinfo.konkuk.ac.kr/INTERSPIA/	protein–protein interactions in multiple species
**iPath3.0**	http://pathways.embl.de	metabolic pathway visualization and customization
**IUPred2A**	http://iupred2a.elte.hu	intrinsically disordered protein regions
**Kinact**	http://biosig.unimelb.edu.au/kinact/	kinase activating missense mutations prediction
**KnotGenome**	http://knotgenom.cent.uw.edu.pl/	topological analysis of chromosome knots and links
**LitVar**	https://www.ncbi.nlm.nih.gov/CBBresearch/Lu/Demo/LitVar	genetic variant information retrieval from PubMed
**LOLAweb**	http://lolaweb.databio.org	genomic region enrichment analysis
**MetaboAnalyst 4.0**	http://metaboanalyst.ca	metabolomics data analysis
**MetExplore**	https://metexplore.toulouse.inra.fr/metexplore2/	metabolic network analysis
**MiGA**	http://microbial-genomes.org/	prokaryotic genome and metagenome classification
**MISTIC2**	https://mistic2.leloir.org.ar	residue pair covariation in protein families
**MOLEonline**	https://mole.upol.cz	biomolecule channels, tunnels, and pores
**mTM-align**	http://yanglab.nankai.edu.cn/mTM-align/	protein structure multiple alignment and database search
**Mutalisk**	http://mutalisk.org	somatic mutations correlation with genomic, transcriptional and epigenomic features
**Ocean Gene Atlas**	http://tara-oceans.mio.osupytheas.fr/ocean-gene-atlas/	marine plankton gene geolocation and abundance
**oli2go**	http://oli2go.ait.ac.at/	PCR primer and hybridization probe design for non-human DNA
**OmicsNet**	http://www.omicsnet.ca	molecular interactions networks visualization
**oriTfinder**	http://bioinfo-mml.sjtu.edu.cn/oriTfinder	origin of transfer sites in bacterial mobile genetic elements
**PaintOmics 3**	http://bioinfo.cipf.es/paintomics/	visualization of omics data on KEGG pathways
**PANNZER2**	http://ekhidna2.biocenter.helsinki.fi/sanspanz/	protein function prediction
**PatScanUI**	https://patscan.secondarymetabolites.org/	DNA and protein sequence pattern search
**PhytoNet**	http://www.gene2function.de	phytoplankton gene expression profiles
**pirScan**	http://cosbi4.ee.ncku.edu.tw/pirScan/	piRNA target prediction
**ProTox-II**	http://tox.charite.de/protox_II	chemical toxicity prediction
**psRNATarget**	http://plantgrn.noble.org/psRNATarget/	plant small RNA target prediction
**PSSMSearch**	http://slim.ucd.ie/pssmsearch/	protein motifs for binding and post-translational modification
**PUG-REST**	https://pubchemdocs.ncbi.nlm.nih.gov/pug-rest	PubChem cheminformatics programmatic access
**RepeatsDB-lite**	http://protein.bio.unipd.it/repeatsdb-lite	tandem repeats in proteins
**RNApdbee 2.0**	http://lepus.cs.put.poznan.pl/rnapdbee-2.0/	RNA secondary structure annotation
**RSAT**	http://www.rsat.eu/	DNA regulatory motifs
**SMARTIV**	http://smartiv.technion.ac.il/	RNA sequence and structure motifs for RNA binding proteins
**SNPnexus**	http://www.snp-nexus.org	SNP functional annotation
**SPAR**	https://www.lisanwanglab.org/SPAR	analysis of small RNA sequencing data
**SWISS-MODEL**	https://swissmodel.expasy.org	structure homology modeling for proteins and protein complexes
**TAM 2.0**	http://www.scse.hebut.edu.cn/tam/	microRNA set enrichment analysis
**TCRmodel**	http://tcrmodel.ibbr.umd.edu/	T cell receptor structure modeling
**UNRES**	http://unres-server.chem.ug.edu.pl	coarse-grained simulation of protein structure
**VarAFT**	http://varaft.eu	disease-causing variants annotation
**WEGO 2.0**	http://wego.genomics.org.cn	Gene Ontology visualization
**X2K Web**	http://X2K.cloud	kinase enrichment analysis for differentially expressed gene signatures
**xiSPEC**	http://spectrumviewer.org	proteomics mass spectrometry data analysis

**Topics**. Papers in this year’s issue fall into several broad categories: (i) DNA shape and chromatin conformation, (ii) coding and non-coding RNAs, (iii) gene expression, gene set enrichment and genome interval enrichment, (iv) protein structure, binding, and docking, (v) motifs and pattern search, (vi) microbiome and metagenome, (vii) metabolic networks, (viii) gene and protein function prediction and prioritization of genetic variants, (ix) data visualizations and (x) miscellaneous topics.

In the microbiome and metagenome category, papers include **MiGA**, for bacterial and metagenome classification, the **Ocean Gene Atlas**, which analyzes abundance and distribution of marine plankton genes, and **BAGEL4**, for identification of secondary metabolite gene clusters including bacteriocins and ribosomally synthesized and posttranslationally modified peptides (RiPPs).

The papers in the protein structure, binding, and docking category include the highly used website **SWISS-MODEL**, for protein structure homology modeling, **BeStSel**, for protein secondary structure analysis from circular dichroism spectroscopy data, **UNRES**, for coarse-grained simulation of protein structure and dynamics, **CABS-flex 2.0** for simulation of protein structure flexibility, **IUPred2A**, for predicting intrinsically disordered protein regions, **InterEvDock2** for protein–protein docking, **ComplexContact** for modeling protein heterodimer complex residue–residue contacts, **HSYMDOCK**, for modeling docking in symmetric protein complexes, **HPEPDOCK**, for peptide-protein docking, **AlloFinder**, which seeks to identify allosteric modulators of protein–ligand binding, and **HotSpot Wizard 3.0**, for protein engineering using directed mutation.

Tools for RNA analysis include **Freiburg RNA tools**, a broad collection of RNA analysis tools, **pirScan**, for predicting targets of PIWI interacting RNA (piRNA) and **psRNATarget**, for plant small RNA target analysis.

Tools for analysis of metabolic networks include **MetaboAnalyst 4.0**, an update to the popular website for analysis of metabolomics data, and **MetExplore**. Several papers in the visualization category also deal with metabolic networks, including **PaintOmics 3**, for visualization of omics data on KEGG pathways and **iPath3.0**, for visualization and annotation of metabolic pathways.

Function prediction tools include an update to the popular **GeneMANIA** gene function prediction website, **GIANT 2.0**, for tissue specific gene function prediction and **PANNZER2**, for protein function prediction.

Highlights among the remaining papers include an update to the widely used **GALAXY** workflow platform, which contains an extensive set of tools for reproducible biological data analysis, **CircadiOmics**, which provides analysis of omics data for circadian rhythm analysis, **cgDNAweb**, which provides structural models of double stranded DNA fragments, **ProTox-II**, which predicts small molecule chemical toxicity, **xiSPEC**, for proteomics mass spectrometry data analysis, a text mining entry, **ezTag**, for semi-automated biomedical concept annotation, **CRISPOR**, for design of CRISPR/Cas9 guide sequences, an update to the **HMMer** website for homology search using profile hidden Markov models, and a 20th anniversary paper on **RSAT**, which detects DNA regulatory motifs.

**Stand-alone programs and web services**. The web server issue additionally has special sections for stand-alone tools for high-throughput data analysis, which must be installed on the researcher’s computer, and web services for data and analysis, which are accessed programmatically rather than by manual interaction with a website. One paper, **PUG-REST**, reports on programmatic access to the PubChem cheminformatics data and services. Two papers report on stand-alone tools, **gRINN**, which analyzes residue interaction energies in protein molecular dynamics simulations, and **VarAFT**, a variant annotation and filtering tool, which works with variant call format (vcf) files.

**Acknowledgements:** The web server issue arises out of the work of many people who I would like to thank. First there are the researchers and scientific programmers who provide us high-quality, freely available web resources and who revise and improve their manuscripts and websites under considerable time pressure. Next are the hundreds of referees who conscientiously contribute their time to reviewing and helping improve the manuscripts and websites.

**Figure 1.**
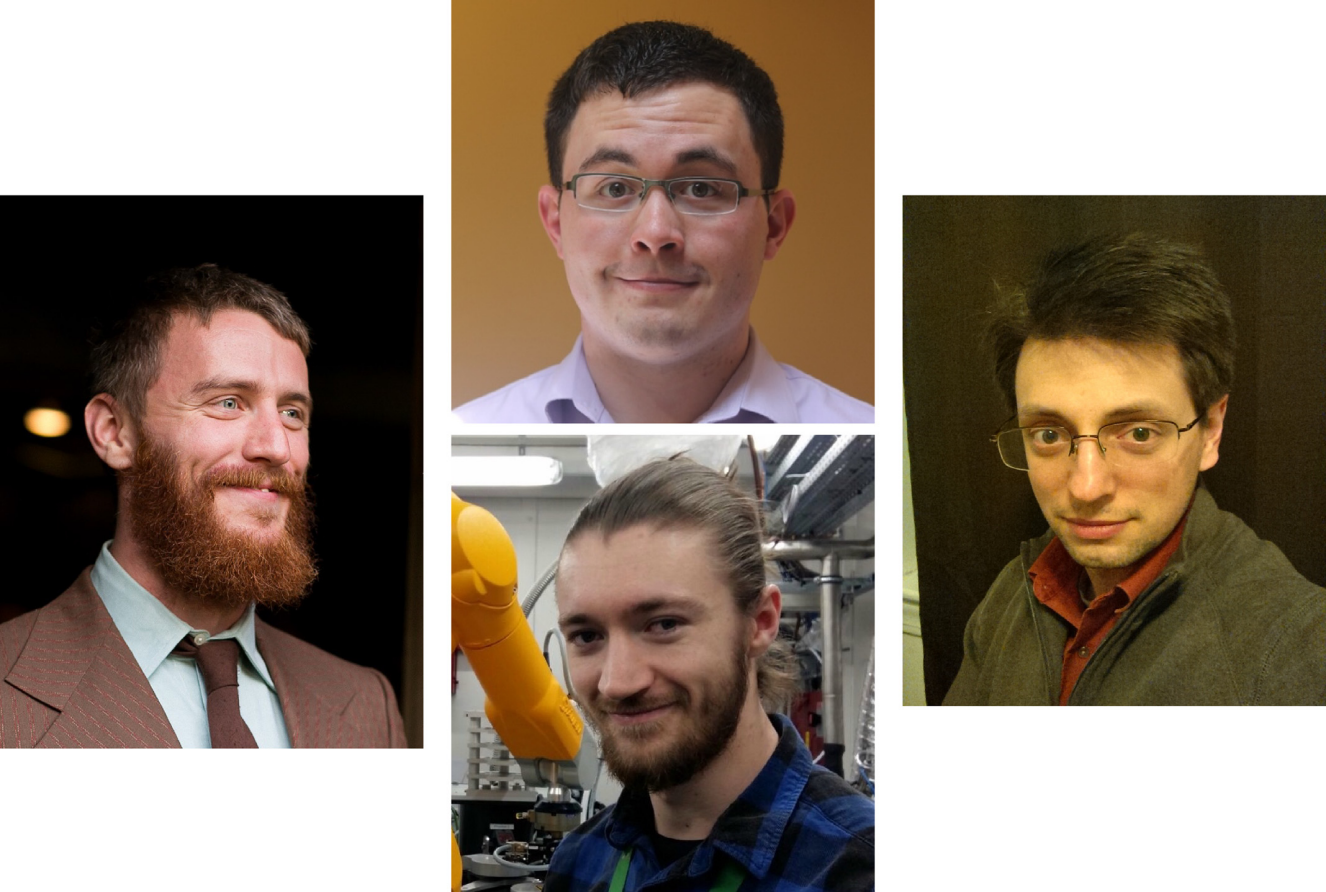
Clockwise from left: Sean Corbett, David Jenkins and Tyler Faits are PhD students in the Boston University Bioinformatics Graduate Program. Adam Simpkin is a PhD student at the Institute of Integrative Biology, University of Liverpool, UK/Synchrotron SOLEIL, France. They provided outstanding assistance in testing the web server proposal websites.

My own work is made enormously easier by the dedicated editorial assistance of Fay Oppenheim, who logs the data for all the proposals and interacts with the referees, inviting, cajoling, and chasing them so that their reviews are returned on time. Thank you. Thanks also to Sean Corbett, Tyler Faits, and David Jenkins, PhD students in the Boston University Bioinformatics Program, and Adam Simpkin, PhD student at the Institute of Integrative Biology, University of Liverpool, UK/Synchrotron SOLEIL, France, for their hard work in carefully evaluating the proposal websites during the proposal approval phase (Figure 1). Additional thanks to Martine Bernardes-Silva, Editorial Manager, *NAR*, and Joanna Ventikos and the staff at Oxford University Press.

**Deadlines for 2019**. To streamline the review process, authors are required to send a one-page summary of their web server to the editor, Dr Gary Benson (narwbsrv@bu.edu), for pre-approval prior to manuscript submission. Authors should consult the instructions for summary submission and website design at https://academic.oup.com/nar/pages/submission_webserver. For 2019, the summary and URL address of the fully functional website must be submitted by 31 December 2018. The deadline for submission of articles is 31 January 2019.

**Instructions for submissions**. Review of a proposal includes evaluation of the summary and extensive testing of web server functionality. The key criteria for approval are high scientific quality, wide interest, ability to do computations on user-submitted data, and a well-designed, well-implemented, and fully functional website. Note that there is a minimum two-year interval before publication in the web server issue for websites, or essentially similar websites, that have been the subject of a previous publication, including in journals other than NAR. With respect to the website, the following are guidelines for approval.

1. It should have an easy-to-find submission page with a simple mechanism for loading test data and setting test parameters. The preferred method is one-click loading. The test data should be available for download so that users can examine the data format. Additional mechanisms that assist the user in submitting data should be implemented where appropriate, for example, automatic loading of a pdb structure file once the user has entered the appropriate identifier.
2. Output should be dynamic and rich in detail. Wherever possible, supporting evidence used in calculations and/or links to external databases containing additional information should be provided. Numerical, textual, and graphical output should be mixed and visualization tools that add information or increase the user's understanding should be utilized. Note that output consisting merely of a few numerical values, a static spreadsheet, or a series of files to be opened in other programs will not be approved. Note also that for security reasons, use of FLASH and Java plugins will no longer be allowed.
3. Web servers that do not finish their calculations immediately must implement a mechanism for returning results to the user. Notification by email may be provided as an option, but an alternative that returns a web link at the time of data submission, which the user can then bookmark and access at a later time, is required. This link should ideally report the status of the job (queued, running, finished) and an estimate of the overall time for job completion. Websites that require registration and/or a login will not be approved. Note that uploaded data and the results of analysis for each user must be private and not viewable by other users.
4. The website should be supported by an extensive help section or tutorial that guides the user through the submission process, contains details about input file formats and parameters, and explains the meaning of the output. Whenever possible, the help pages should link to dynamic output examples similar to those provided by the website. When video tutorials are used, text and figure help pages should also be available to simplify quick look-up.
5. Any proposal for a web server that is predictive must include details on validation of predictions from new data not used in training. *N*-fold cross validation methods will not be considered sufficient. Details should include size and composition of the validation data set (number of positive and negative cases), and several measures of predictive performance, including sensitivity, specificity, and precision. Proposals are frequently rejected for lack of adequate prediction validation information.
6. Websites not clearly designed to accept and analyze user-submitted data will be rejected. This applies to those established primarily for lookup or exploration in a data set, or serve the function of data aggregators. Authors of websites that provide novel data should consider the NAR Database Issue as a possible venue (see the instructions at https://academic.oup.com/nar/pages/ms_prep_database).
7. Proposals that describe a new analysis method are generally not appropriate for the web server issue because limited space and the rapid revision process make thorough method description and validation problematic. Authors of such methods might instead consider sending their manuscript to *NAR* as a regular computational biology paper (see the instructions for authors at https://academic.oup.com/nar/pages/Criteria_Scope#Computational%20Biology).

Gary Benson

Executive Editor

Web Server Issue

*Nucleic Acids Research*

May 2018

